# Assembly of the Intraskeletal Coral Organic Matrix during Calcium Carbonate Formation

**DOI:** 10.1021/acs.cgd.3c00401

**Published:** 2023-07-15

**Authors:** Silvia Milita, Tal Zaquin, Simona Fermani, Devis Montroni, Iddo Pinkas, Luisa Barba, Giuseppe Falini, Tali Mass

**Affiliations:** † CNR—Institute for Microelectronic and Microsystems, via Gobetti 101, Bologna 40129, Italy; ‡ Department of Marine Biology, The Leon H. Charney School of Marine Sciences, University of Haifa, Mt. Carmel, Haifa 3498838, Israel; § Department of Chemistry “Giacomo Ciamician”, University of Bologna, via Selmi 2, Bologna 40126, Italy; ∥ Interdepartmental Centre for Industrial Research Health Sciences & Technologie, University of Bologna, Bologna 40064, Italy; ⊥ Department of Chemical Research Support, Weizmann Institute of Science, Rehovot 76100, Israel; # CNR -Institute of Crystallography, Elettra Synchrotron, Trieste I-34100, Italy; ¶ CNR, Institute for Nanostructured Materials, via Gobetti 101, Bologna 40129, Italy

## Abstract

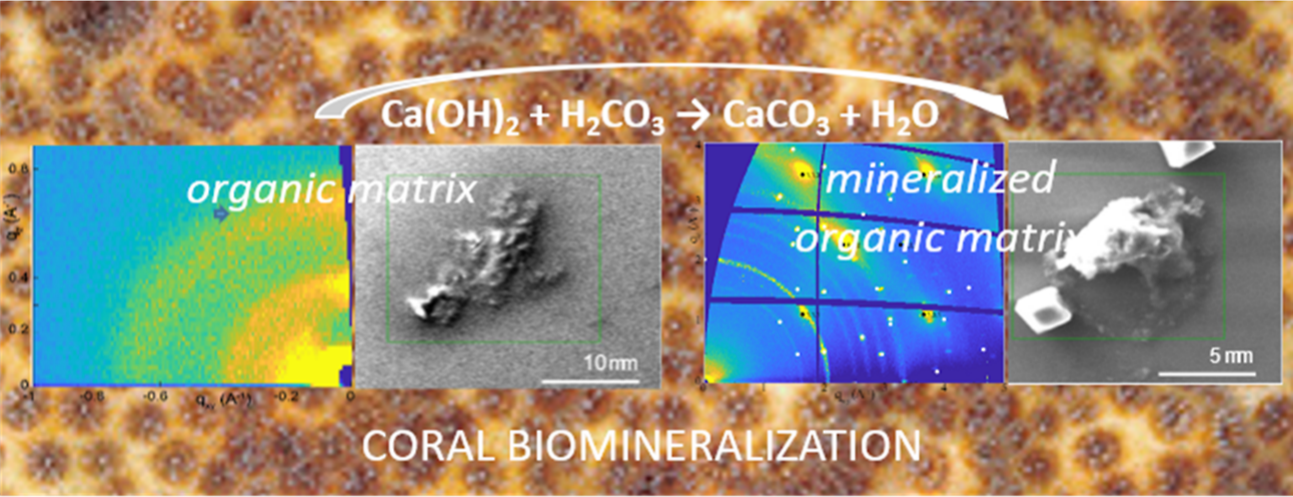

Scleractinia coral skeleton formation occurs by a heterogeneous process of nucleation and growth of aragonite in which intraskeletal soluble organic matrix molecules, usually referred to as SOM, play a key role. Several studies have demonstrated that they influence the shape and polymorphic precipitation of calcium carbonate. However, the structural aspects that occur during the growth of aragonite have received less attention. In this research, we study the deposition of calcium carbonate on a model substrate, silicon, in the presence of SOM extracted from the skeleton of two coral species representative of different living habitats and colonization strategies, which we previously characterized. The study is performed mainly by grazing incidence X-ray diffraction with the support of Raman spectroscopy and electron and optical microscopies. The results show that SOM macromolecules once adsorbed on the substrate self-assembled in a layered structure and induced the oriented growth of calcite, inhibiting the formation of vaterite. Differently, when SOM macromolecules were dispersed in solution, they induced the deposition of amorphous calcium carbonate (ACC), still preserving a layered structure. The entity of these effects was species-dependent, in agreement with previous studies. In conclusion, we observed that in the setup required by the experimental procedure, the SOM from corals appears to present a 2D lamellar structure. This structure is preserved when the SOM interacts with ACC but is lost when the interaction occurs with calcite. This knowledge not only is completely new for coral biomineralization but also has strong relevance in the study of biomineralization on other organisms.

## Abstract 2

The intraskeletal organic matrix from the coral skeleton changes its assembly during the in vitro formation of calcium carbonate.

## Introduction

Many biomineralization processes occur on a structured substrate which can be a crystalline plane or an array of macromolecules.^1,2^ Thus, the growth of a biomineral, the composite inorganic–organic material produced by organisms, is generally a heterogeneous process. A widely studied biomineral is calcium carbonate, which is the main mineral phase associated with invertebrates.^1^ In this context, examples of calcium carbonate biominerals are coral skeletons,^3–5^ mollusk shells,^6,7^ foraminifera shells,^8^ coralline red algae,^9^ echinoderm endoskeleton, and teeth.^10,11^

Recent studies indicated that in many cases, the growth of biogenic calcium carbonate occurs through the initial formation of amorphous calcium carbonate (ACC) particles that crystallize once anchored to a substrate.^12–14^ A series of molecular dynamic simulations showed that the ions on the surface of ACC particles have a higher mobility than their bulk counterparts.^15^ This mobility allows them to preferentially interact with acidic amino acids, suggesting that crystallization occurs in this region via solid-state transformation.^16,17^ This discovery was experimentally confirmed by applying a combination of high-resolution imaging and in situ solid-state nuclear magnetic resonance spectroscopy.^15^ Accordingly with these experiments, the classic theory of crystal nucleation and growth does not apply to biominerals.^18^ An important consequence is that one of the experimental proved biomineralization paradigms, i.e., the epitaxial growth of a mineral phase on a macromolecular template,^19–23^ seems to need a deeper investigation. Furthermore, the interaction between the ACC particles and the heterogeneous substrate at the atomic and molecular levels has been poorly investigated.

Grazing incidence X-ray diffraction (GIXD) is a technique extensively employed in the study of surfaces of inorganic single crystals, crystal nucleation, and thin films (monolayers and few layers thick) deposited on a flat surface. In GI, the scattered intensities are recorded when the X-ray beam impinges on the surface with a very small incident angles, α_i_, close to the critical angle for the total external reflection α_c_. As such, the proper choice of the α_i_, allows one a depth resolved investigation of the structural properties, due to the dependence of the X-ray penetration depth on the α_i_. When α_i_ < α_c_ only the surface layers (few Å thick) are probed, while for α_i_ > α_c_ several micrometers are investigated. In GI geometry, both the signals scattered at wide (GIWAXS) and small angles (GISAXS) can be recorded, in the GIWAXS or GISAXS techniques, respectively, providing information on periodicities in the order of Å and nm. Diffraction patterns, recorded at wide angles, can be obtained from crystalline structures on surfaces,^24^ and in particular, they allow Dutta and co-workers to demonstrate a 1:1 match between the arachidyl sulfate monolayer unit cell and the unit cell of the (001) plane of calcite.^25^ This showed that the GIWAXS techniques can be efficiently applied for the study of calcite growth along preferential orientations.

In situ GISAXS was used to measure the nucleation rates at different supersaturations of CaCO_3_, further providing thermodynamic parameters for subsurface reactive transport modeling regarding CaCO_3_ formation on surfaces.^26^ Plaster samples biotreated were analyzed by microprobe X and GIXD. The results showed only the presence of calcite, no transitory state was observed.^27^ X-ray reflectivity (XRR) is a complementary surface technique that produces electron density profiles as a function of distance to the interface and can also provide information on nondiffracting materials.^24^ DiMasi and co-workers used XRR to distinguish between the different kinetic effects of polymer, magnesium ions, and mineral ions concentrations on the formation and stabilization of ACC under a fatty acid monolayer.^28^ The combination of GIXD and XRR measurements for the study of CaCO_3_ provides information about the structure of the substrate molecules and crystals formed. This showed that the conformational plasticity of the surface molecules leads to the nucleation of calcite with growth along different crystallographic orientations.^29^

It is reported in literature that the coral biomineralization process occurs by the heterogeneous formation of aragonite fibers on Mg-calcite on which intraskeletal organic molecules are adsorbed.^30,31^ Recent studies characterized the soluble fraction (SOM) composition and structure of the intraskeletal organic molecules extracted from different coral species, *Stylophora pistillata* and *Oculina patagonica*.^32^ These studies showed that the SOM induced the overgrowth of calcium carbonate on Mg-calcite with different textures in the absence of Mg ions.^33^ Here, we further aim to test the role of coral SOM in calcium carbonate deposition. We hypothesize that the presence of SOM adsorbed on a substrate modifies the deposition of calcium carbonate at the molecule–mineral interface, and we aim to study this process by GIWAXS, optical microscopy, scanning electron microscopy (SEM), energy-dispersive X-ray spectroscopy (EDX) mapping, and micro-Raman spectroscopy. To properly perform the experiment, we chose a silicon wafer as substrate on which to adsorb the SOM. The chemical inertness and high flatness of this material is required to accurately control α_i_. This substrate does not have relevance in coral biomineralization, where is well-known that mineral deposition starts on the surface of coralline algae and continues on magnesium calcite and aragonite.^34^ However, it is the best one to show the potentiality of the GIWAXS techniques, which poses the constraints reported above. The deposition of calcium carbonate was induced using a calcium hydroxide solution and a carbonic acid solution, avoiding the formation of salt coproducts. The SOMs extracted from the skeletons of *S. pistillata* and *O. patagonica* were used. We proposed to study the fast-growing tropical coral *S. pistillata* and the slow-growing temperate coral *O. patagonica*. Those corals represent different growth strategies and diverse growth conditions and, in addition, in a recent study we characterized the SOM of both species.^32^

## Experimental Section

### Preparation of CaCO_3_ Dispersion

Calcium carbonate was synthesized by mixing equal volumes (300 μL) of both calcium hydroxide and carbonic acid solutions. The latter solution was prepared by bubbling a high-grade carbon dioxide gas into water until a constant pH (3.99) was achieved. The concentration of this solution was then determined by titration using a standard NaOH solution (*c* = 0.10 M). Calcium hydroxide solution was prepared by adding an excess of calcium hydroxide to water. The suspension was stirred for 3 h and then filtered through a 0.22 μm membrane filter, both processes occurred under an atmosphere of nitrogen. The concentration was determined by titration using the standard HCl solution (*c* = 0.10 M). In all the experiments, Milli-Q water (conductivity <0.1 mS cm^–1^) was used. The freshly prepared carbonic acid solution, pure or with the required amount of dissolved SOM was always poured into the calcium hydroxide solution.

### SOM Extraction

Approximately 2.5 g of powdered skeleton was treated overnight with 200 mL of sodium hypochlorite (5 vol %) solution. Successively the powder was washed with water and lyophilized. The surface cleaned skeleton powder was used from each sample to extract the coral skeletal soluble organic macromolecules (SOM) using the method described in Reggi et al. (2020).^35^ In brief, powdered samples were poured into a dialysis bag (MWCO 3500 Da) with a small volume of water. The dialysis bag was immersed in a 0.1 M acetic acid solution until complete dissolution of the mineral phase. Samples were then centrifuged at 5000*g* for 5 min at 4 °C, and the supernatant was collected and lyophilized. The SOM concentration (μg/mL) was expressed as the amount of protein from the amino acid analysis.

### Silicon Wafer Substrate Preparation

SiO_2_/Si substrates 1 × 1 cm^2^ were sequentially sonicated in DI water, acetone, and isopropyl alcohol for 10 min each.

### Deposition of SOM CaCO_3_ and CaCO_3_/SOM Mix on Silicon Wafer Substrates

Freshly prepared 300 μL of the CaCO_3_ suspension was kept in a plastic vial for a time ranged between 0 and 15 min (*t*_mix_). After this period, 100 μL of the suspension was transferred onto the surface of the substrate and left there for a *t*_cast_ time ranging between 0 and 10:00 min. Finally, the dispersion was spin-coated at 2000 rpm for 2:00 min. These experimental conditions are summarized in Table S1.

### Optical Microscopy Observations

Optical microscopy images were collected in the reflection mode using a Leica DM4000 optical microscope. The images were also collected by using crossed-polarizers to check for the presence of birefringence.

### SEM Analyses

The SEM images were collected on samples without any coating by using a ZEISS Evo LS10 under low pressure operating at 30 kV. EDS spectra and maps were recorded using an X-rays EDS spectrometer Bruker Quantax 200 × 30 mm^2^.

### Micro-Raman Spectroscopy Measurements

Raman measurements were conducted on a LabRAM HR Evolution instrument (Horiba. France). The instrument is equipped with an 800 mm spectrograph which allows for sub-two wavenumber pixel spacing when working with 1800 grooves/mm grating at 325 nm excitation. The sample was exposed to the laser light by an ×40 Near UV NA = 0.47 objective (LMU-40×-NUV, Thorlabs. USA). The LabRAM instrument has a 1024 × 256 pixel open-electrode, front-illuminated, and cooled CCD camera. The system is set around an open confocal microscope (BX-FM Olympus. Japan) with a spatial resolution better than 2 μm by using the 40× Near UV objective. Exposure was set according to the signal intensity, which required exposure between 15 s and 1 min. This system is equipped with ultralow frequency capability, four laser lines, many objectives, and several gratings to allow modular and flexible use for samples of significant variability.

### Grazing Incidence Diffraction Measurements

GIWAXS measurements were performed at the XRD1 beamline of the ELETTRA, a synchrotron radiation facility in Trieste, Italy. A Pilatus X-ray detector (DECTRIS Ltd. Baden, Switzerland) was used to collect the 2D images. The sample detector distance was 200 mm. The wavelength was fixed to λ = 0.7 Å and the beam size was of 200 × 200 μm^2^. Different X-ray beam incident angles α_i_ were selected, below and above the critical angle for the total reflection (0.07°), to probe the surface layer (few nm) and full film thickness (the X-ray penetration depths are ∼1000 nm).

## Results and Discussion

### Experimental Setup

**Figure 1:**
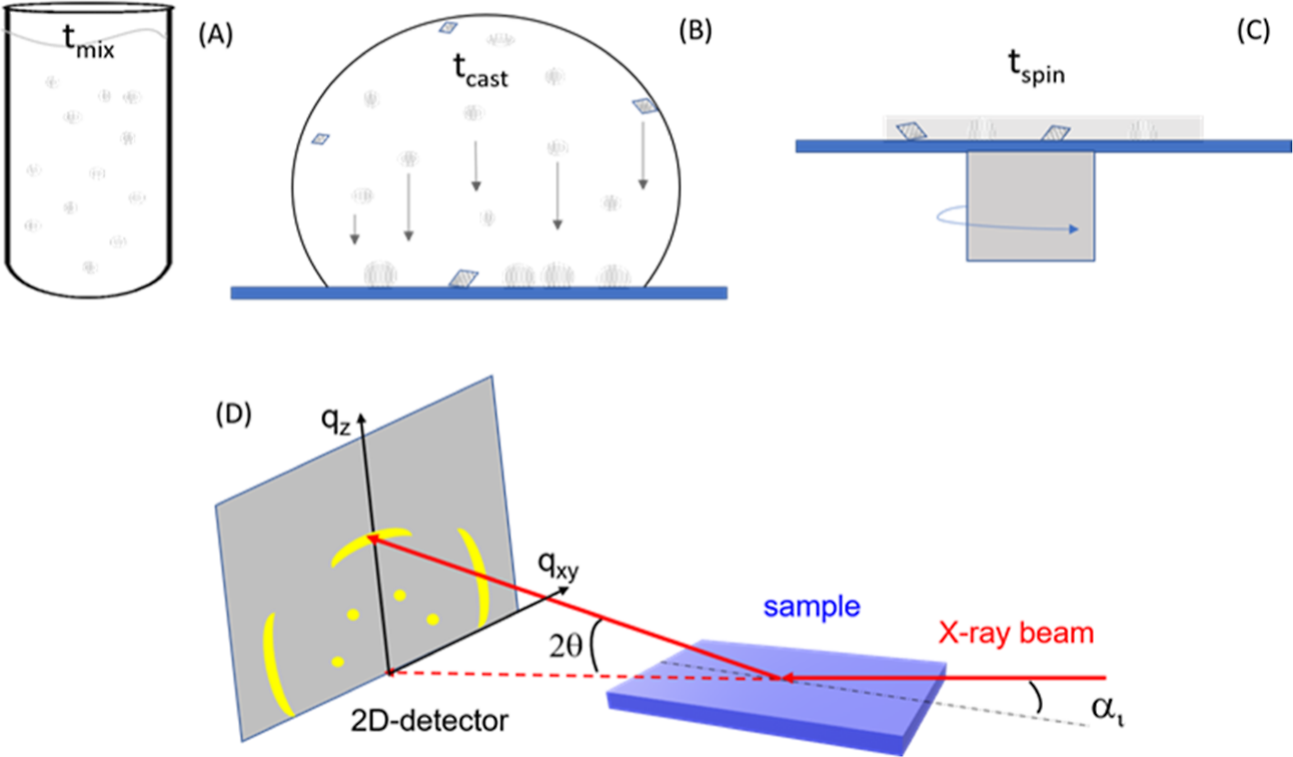
Sequential different stages of sample preparation. (A) Mixing of the reactants, calcium hydroxide, and carbonic acid; *t*_mix_ indicates the time of mixing. (B) Deposition of the CaCO_3_ particles on the substrate for a time *t*_cast_. (C) Spin coating process to leave a thin layer on the surface. The circles indicate ACC particles and prenucleation clusters, while the rhombuses indicate CaCO_3_ crystalline particles. (D) Sketch of the GIWAXS setup. The dots and arcs on the 2D detector represent diffraction signals from highly and weakly oriented crystallites, respectively.

In this research, a crucial initial step was the definition of the experimental setup. The choices were made having in mind the following requests: (i) the formation of prenucleation cluster or crystalline phase precursors should occur in solution; (ii) the mineral phase should preferentially form on the substrate; and (iii) only the formation of a calcium carbonate mineral should take place. To achieve these goals, as the first choice, we precipitated CaCO_3_ by mixing a solution of calcium hydroxide with a solution of carbonic acid.^36^ This CaCO_3_ precipitation method has been already used in in vitro studies of biomineralization processes where SOM, peptides and several ions have been used as additives.^36–38^ When equal volumes (300 μL) of 1.2 × 10^–2^ M H_2_CO_3_ solution and 1.0 × 10^–2^ M Ca(OH)_2_ solution were mixed, a milky dispersion appeared. The used concentrations are not related to the concentration of Ca and carbonate ions in seawater. They were mainly selected by the experimental set-up and by the requirement to be above the saturation limit of ACC.^39^ This was because several reports indicate that the biomineralization of corals occurs through a transient ACC phase.^40^ The initial supersaturation is defined as relative supersaturation, *S* – 1, *S* = (∏/*K*_sp_)^1/2^, where ∏ is the ion activity product *a*(Ca^2+^)·*a*(CO_3_^2–^), and *K*_sp_ is the thermodynamic equilibrium constant of dissolution of the most stable calcium carbonate polymorph, calcite (*K*_sp_ = 3.3 × 10^–9^ at 25 °C). From the known total concentrations of Ca(OH)_2_ and H_2_CO_3_ initially mixed, the molar concentrations and the corresponding activities of all relevant ionic species that were assumed to exist at considerable concentrations in the solution (H^+^, OH^–^, CO_3_^2–^, HCO_3_^–^, CaCO_3_^0^, CaHCO_3_^+^, Ca^2+^, and CaOH^+^) were calculated. The detailed calculation procedure, which takes into account the respective proteolytic equilibria and equilibrium constants as well as the charge- and mass-balance equations, has been described previously.^41^ The used concentrations allowed to have a mixture with an initial CaCO_3_ supersaturation with respect to calcite of 22.2 (pH = 9.8) and most importantly, highly supersaturated with respect to all the crystalline polymorphs of CaCO_3_ and ACC, hence allowing their formation by a spontaneous precipitation process.^39^ In this condition of high supersaturation, the deposition of CaCO_3_ is governed by kinetic processes, which can be defined by the following experimental parameters: the time of mixing (*t*_mix_), the time of casting (*t*_cast_), and the time of spinning (*t*_spin_). *t*_mix_ represents the period of time that spams from the mixing of the reactants (i.e., calcium hydroxide and carbonic acid solutions) to the deposition on the substrate of a volume (100 μL) of the CaCO_3_ dispersion. During this time, due to the high starting supersaturation, prenucleation clusters form, some crystalline nuclei may form as well.^42^ The deposited volume of CaCO_3_ dispersion, when observed under an optical microscope with crossed-polarizers (Figure S1), does not show any birefringence for a time shorter than 2 min. Thus, most of the milky material is CaCO_3_ and has no optical microscope detectable crystalline state. *t*_cast_ represents the time in which a volume of the CaCO_3_ dispersion is kept on the substrate. During this time, some prenucleation clusters and crystal nuclei continue to form, and eventually small crystals deposit on the substrate, on which nucleation and growth can also occur. *t*_spin_ is the spinning time, during this process part of the solution is expelled from the substrate, the solvent completely evaporates and a layer of calcium carbonate forms on the substrate. This layer also contains the CaCO_3_ particles that formed during the casting time. The volume of dispersion that is expelled by the substrate during the spinning process is not quantifiable, but it is reasonable to suppose that it represents most of the volume casted on the substrate. Aiming to have on the substrate mostly the deposition of ACC before the spinning process, and in a quantity high enough to be detected by GIWAXS, a series of optimization experiments was performed (Table S1). A volume of CaCO_3_ dispersion of 100 μL, a *t*_mix_ of 1:00 min, and a *t*_cast_ of 2:30 min were used. A *t*_spin_ of 2:00 min allowed for a dry precipitate on the substrate a dry precipitate. The overall process of CaCO_3_ deposition is illustrated in Figure 1, which also reports a sketch of the GIWAXS setup.

Two typologies of experiments were performed, one having the SOM adsorbed on the silicon wafer before CaCO_3_ deposition (sequential) and one having the SOM in solution mixed with the CaCO_3_ dispersion (mix). The materials deposited on the silicon wafer, along with the GIWAXS experiments, were characterized by optical microscopy, SEM, EDX mapping, and Raman microscopy. Comparing the results from these two typologies of experiments, we faced some limitations given by the experimental setup used. The amount of material, CaCO_3_ and SOM, deposited on the substrate can be different. Indeed, in the sequential deposition of SOM and CaCO_3,_ the deposition of CaCO_3_ mainly occurs during the *t*_cast_, as discussed above, whereas a part of the SOM could dissolve in the CaCO_3_ dispersion. However, we are confident from the experimental observations that this fraction of SOM is very low since SOM has the capability to interact with CaCO_3_ making precipitates.^33^ In the mixing system, the capability of the SOM and the CaCO_3_ to interact with the substrate can be different, and thus, a different amount of material could be deposited. We considered that the quantification of the final SOM deposited on the substrate using the two different experimental approaches may not represent the material that we are analyzing by GIWAXS due to the inhomogeneity of the precipitate coverage of the substrate evidenced by optical and electron microscopies (see here coming results). However, despite these limitations, which cannot be avoided, the following results are relevant in comparing the behavior of the SOMs from different species in the intra- and inter-experimental setups.

### Control and Reference Experiments

**Figure 2:**
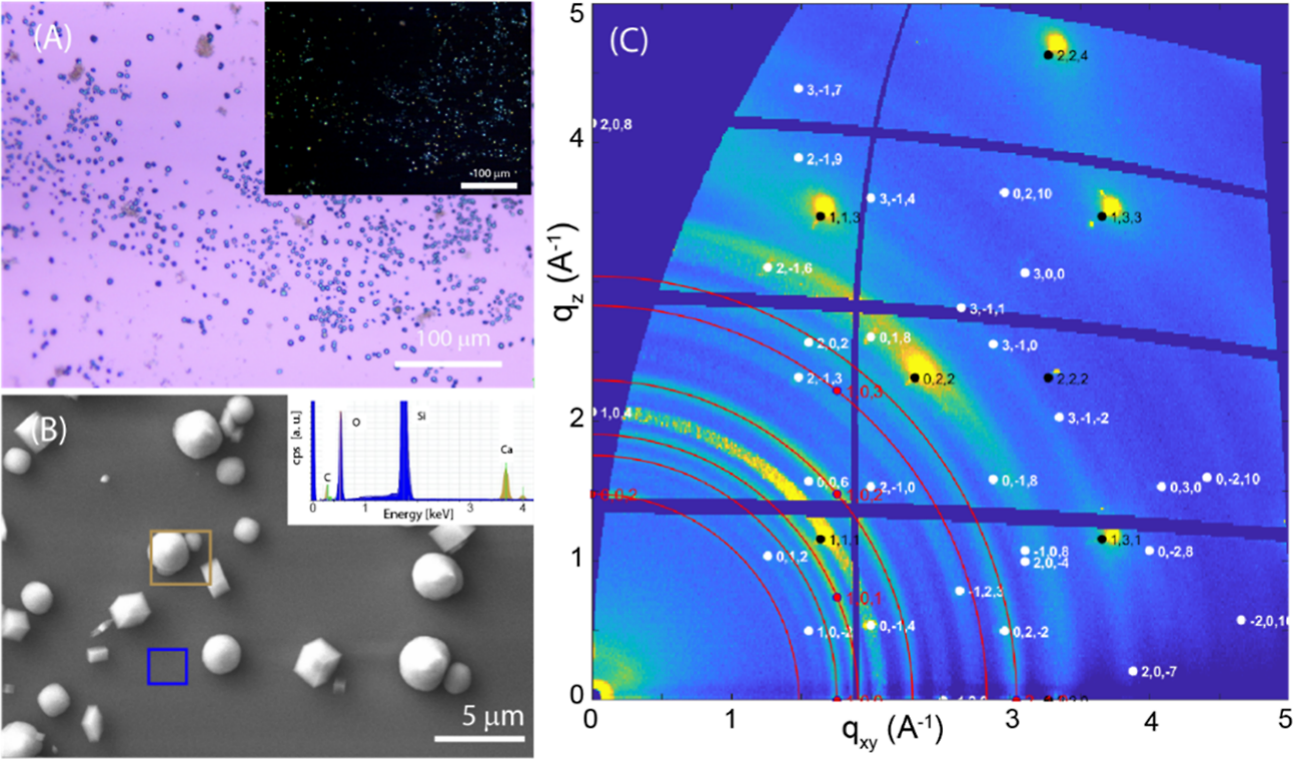
Control experiment on formation of CaCO_3_ on the surface of a silicon wafer without SOM added. (A) Optical microscopy image and (inset) crossed-polarizer optical microscopy image. (B) SEM image of the sample. The inset reports the EDX spectra of the regions inside the squares according to the colors. (D) 2D-GIWAX image in which the red Debye rings indicate the diffraction effects from vaterite, the white spots are the Bragg peaks from calcite and those black from the silicon substrate. All the diffraction peaks come from vaterite, calcite, and silicon substrate and were indexed according to crystalline structure.^43^

A control experiment using only the CaCO_3_ dispersion and in the absence of SOM was performed. It showed the nonuniform deposition of the material (Figure 2A), which is crystalline, as deduced by the birefringence visible in crossed-polarizers optical images (Figure 2A inset) and arranged in rhombohedral and spherulitic crystals (Figure 2B), the typical morphology of calcite and vaterite phases, respectively. The regions of the substrate among the crystals are not only free of crystalline material, being not birefringent (Figure 2A inset), but also do not contain any CaCO_3_ materials, as is visible in the EDX spectrum free of signals from calcium and carbon (Figure 2B inset). The presence of the two phases has been confirmed by the analysis of the 2D-GIWAXS images, where the reflections of calcite (marked in white) and vaterite (in red) phases have been indexed. Signals from the underneath Si substrate are marked in black.

**Figure 3:**
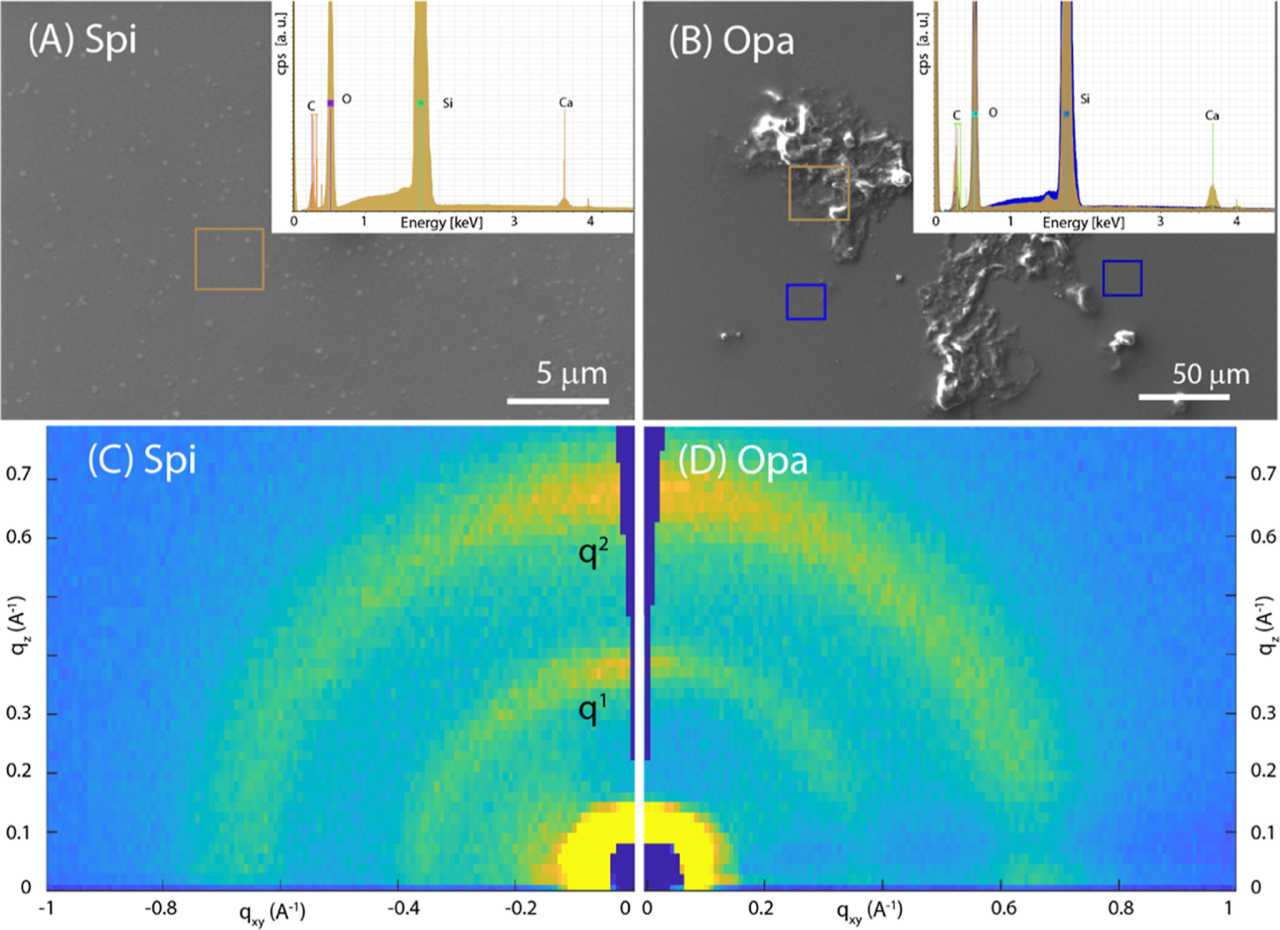
(A,B) SEM images of SOM spin-coated on a silicon wafer from *S. pistillata* (Spi) and *O. patagonica* (Opa), respectively. In the inset the EDX spectra recorded from the square regions drawn on the SEM images are reported. (C,D) 2D-GIWAXS images from Spi and Opa, respectively, deposited on the substrate. *q*_^1^_ and *q*_^2^_ indicate the diffraction peaks analyzed in Table 1.

The profile of the diffracted intensity integrated over the entire 2D-GIWAXS image (Figure 2A) versus the *q* vector is reported in Figure S2 (black line). A quantitative analysis (for instance by using the Rietveld method^44^) is not possible due to the diffracted signals coming from polycrystalline materials (vaterite) and single crystals (calcite). However, taking into account that vaterite reflections have scattering factors lower than those of calcite,^45^ the diffraction profile (Figure S2) suggests a higher content of vaterite than calcite. This agrees with the distribution of spherical (vaterite) and rhombohedral (calcite) particles observed in the optical and SEM images (Figures S1 and S2). The intensity distribution on the 2D image of the diffraction signals of the two polymorphs provides additional structural information, which highlights the marked difference in their arrangement on the substrate, in agreement with their different morphologies, as depicted by the SEM image (Figure 2). The intensity distribution of diffracted vaterite reflections indicates the presence of polycrystallites having (001) planes preferentially oriented parallel to the substrate. This suggests that the spherulitic crystals, imaged by SEM, consist of crystallites that may nucleate and grow at the substrate surface, which induces their preferential orientation (Figure S3). This preferential orientation was recently observed in vivo at Lake Sturgeon otolith formation and in synthetic crystals, where vaterite spherulites formed by crystallites.^46^ This contrasts with the more common spherulitic growths associated with radiating or concentric textures.^47^ On the other hand, the distribution of the spotty diffraction signals of calcite indicates that it forms crystallites having the (104) face mainly parallel to the silicon surface, together with a small fraction of small crystallites almost randomly oriented, which produce weaker almost uniform (ring) signals. From these data, we cannot infer if this crystallite orientation is induced by the substrate presence or by calcite morphology, being the (104) faces the most prominent ones.^48^ As additional control experiments, the SOM from each of the two species was spin-coated on the silicon substrate before interaction with the CaCO_3_ dispersion. The characterization of these substrates is illustrated in Figures 3 and S4–S7.

The SEM images show that the Spi SOM deposits form an almost continuous homogeneous layer on the Si substrate, while the Opa SOM aggregates in some regions of the substrate surface forming rough islands of material. These deposits do not show birefringence (Figures S4A,B and S5A,B), suggesting that there are no birefringent crystalline materials. The EDX spectra (Figure 3 insets) indicated the presence of C as the main element and Ca, as expected, since SOMs were extracted from an aragonitic skeleton. The EDX maps on selected area (Figures S6A,B and S7A,B) confirmed that the two SOMs distribute differently on the substrate surface. Despite these differences, the corresponding 2D-GIWAXS images are extremely similar (Figure 3C,D). Both deposited SOMs produce two diffraction arcs, centered in the vertical direction at the same *q*_*z*_ value, corresponding to the same periodicity. These values could be related to the interactions among the polysaccharidic regions of the SOM glycoproteins and proteoglycans.^49–51^

The literature on the diffraction patterns from SOMs is absent, and in general, for glycoproteins and proteoglycans, it is quite poor. In a diverse context, similar periodicities were observed in crystalline bacterial cell surface layers.^52^ As previously observed for cellulose,^53^ the presence of two reflections along the out-of-plane direction in GIWAXS images suggests that two populations of crystallites are present, each with a different plane stacked parallel to the surface substrate. One possibility is that these populations are segregated into separate lamella within the sample. Although these reflections could not be indexed, due to the lack of crystallographic data, for the 3D crystalline structure, we expect complementary reflections to be present away from the out-of-plane direction, at an angle that is consistent with the unit cell structure. This is not observed in the GIWAXS data. We hypothesize that the degree of order of chain packing, strong in the out-of-plane direction, is disordered along the plane of the substrate which leads to a diffraction too weak to be detected, suggesting 2D crystal behavior.^54^ As different plane surfaces can have diverse hydrophobicity and hydrophilicity, the hydrophobic–hydrophobic and hydrophilic–hydrophilic interactions are likely to be important for intercrystallite interactions.

The analysis of the series of 2D images recorded at different incident angles (see the series for Spi SOM on silicon in Figure S8) allows depth-resolved insights into the SOM arrangement. It is interesting to note that for both SOMs, the two diffraction peaks maintain exactly the same values in *q* and fwhm, regardless of the incidence angle (see Figure S9 for *q* and fwhm vs α_i_ for the second peak of Spi SOM on silicon), i.e., *q*_^1^_ = 0.37 Å ^–1^ and *q^2^* = 0.68 Å ^–1^, (corresponding to *d* = 16.98 and 9.24 Å, respectively) and fwhm 0.08 and 0.16 Å ^–1^, respectively. This indicates that the same crystalline order and dimensions of crystallites are maintained along the film thickness (Table 1).

**Table 1:** Data Extracted by the Series of 2D-GIWAXS Recorded at Different α_I_ for Spi SOM, Opa SOM, and CaCO_3_ Mixed with Spi (Spi_mix) and with Opa (Opa_mix)^a^

	*q*_^1^_ (Å^–1^)	fwhm^1^ (Å^–^)	*q*_^2^_ (Å^–1^)	fwhm^2^ (Å^–1^)	*A*^1^	*A*^2^	*A*^2^/(*A*^2^ + *A*^1^) (%)	*A*^s^/(*A*^s^ + *A*^b^) (%)
Spi SOM	0.38	0.08	0.68	0.16	10	33	76	from 5 (α_I l_) to 40 (α_I h_)
Opa SOM	0.38	0.10	0.67	0.16	9	29	76	from 5 (α_I l_) to 40 (α_I h_)
Spi SOM mix	0.38	0.08	0.68	0.16	4	9	70	from 0 (α_I l_) to 20 (α_I h_)
Opa SOM mix	0.34	0.10	0.66	0.18	2	3	60	from 0 (α_I l_) to 13 (α_I h_)

a For the two peaks (see Figure 3) we report the position (*q*), the width (*W*), the integrated area (*A*) and their relative area. From the fit of the azimuthal profiles of peak 2, we determine the two Gaussian contributions [sharp (s) and broad (b), and we report their area ratio from the surface region [lowest α_I_ (α_I l_)] to whole thickness [highest α_I_ (α_I h_)].

However, the analysis of the azimuthal profiles indicates a slight depth dependence of molecular misorientation. The experimental azimuthal profile can be reproduced by assuming two Gaussian contributions, a broad and sharp one, associated with crystallites having lower and higher preferential orientation, respectively, i.e., with an associated mosaicity of ∼50 and ∼14°. For both the SOMs, these values are almost constant along the film thickness; however, the relative amount of the two populations changes along the film thickness: the sharp content, estimated by the percentage area ratio, *A* = *A*_sharp_/(*A*_sharp_ + *A*_broad_)·100, increases from 5% (close to the air interface) to 40% (close the silicon substrate), indicating a more pronounced stacking alignment close to the substrate surface (Figure S9 for *A*_p_ % vs α_i_ for the second peak of Spi SOM on silicon).

### SOM—CaCO_3_ Sequential Chemical System

**Figure 4:**
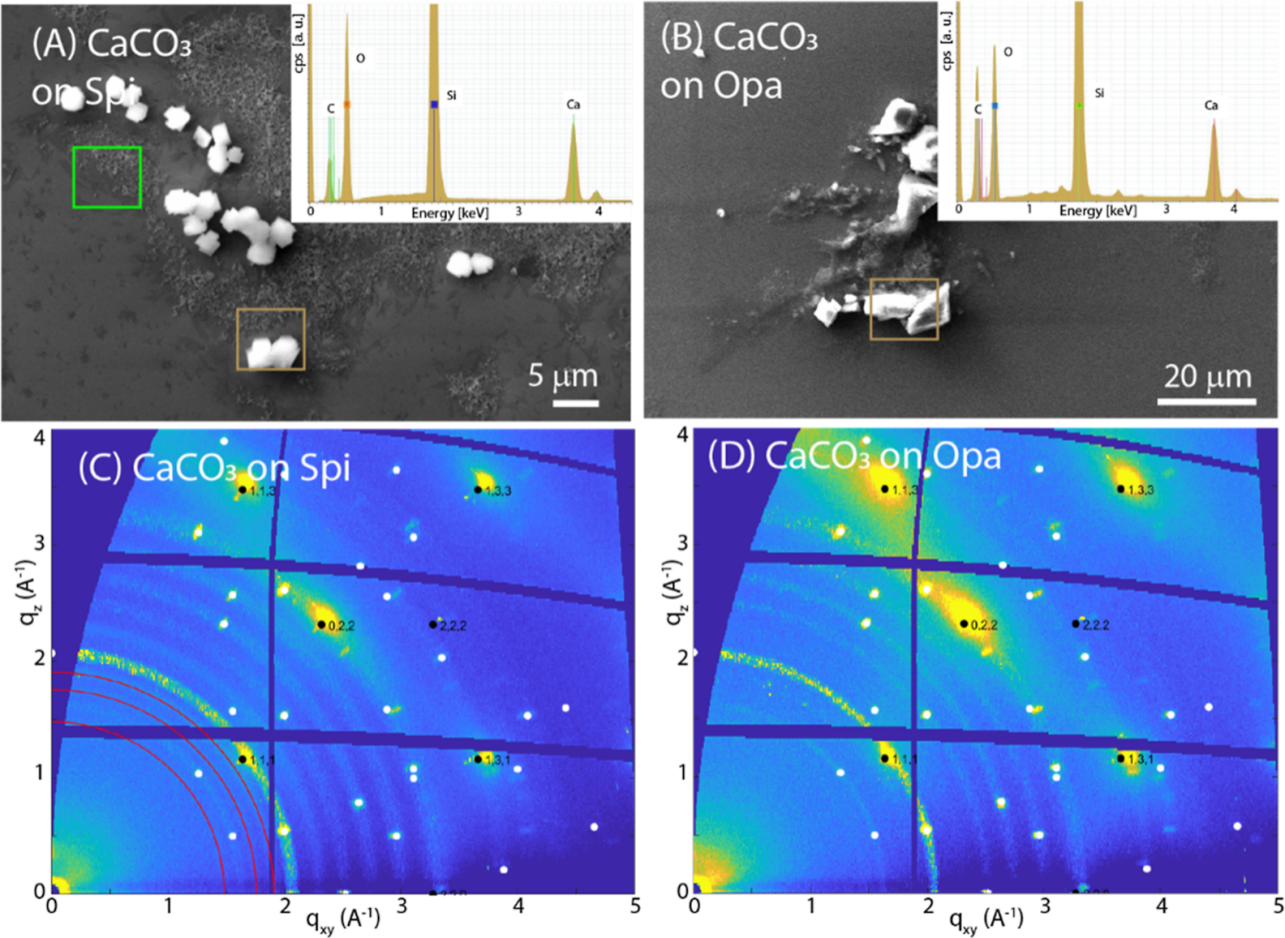
(A,B) SEM images of CaCO_3_ deposited on the SOM layer from *S. pistillata* or *O. patagonica*, respectively. In the inset, the EDX spectra recorded from the square regions drawn on the SEM images are reported. (C,D) GID images from CaCO_3_ deposited on the SOM layer from *S. pistillata* or *O. patagonica*, respectively.

The SOM substrates were used for the templated deposition of CaCO_3_ from the CaCO_3_ dispersion. The crystallite morphologies and structures, as determined by SEM and GIWAXS data (Figures 2–4 and S4–S7) show that the vaterite is largely suppressed (completely in the case of Opa SOM) and mainly the calcite phase forms on the two SOMs deposited substrates, differently from the control in which vaterite was the dominant phase. This result is apparently in contradiction with recent experiments that showed the stabilization of vaterite from SOM macromolecules from the coral skeleton.^33,55^ However, it has to be considered that in one case the vaterite formation was observed using a specific protein of the SOM, the CARP3, in the presence of magnesium ions in solution.^55^ In another case, the vaterite formed on magnesium calcite seeds in the presence of SOM from *S. pistillata*, hence involving the presence of magnesium ions.^33^ In the absence of magnesium ions, it has been reported for a long time that several polyelectrolytes inhibit the formation of vaterite.^56^ SOMs in their protein components are rich in charged Asp and Glu and therefore can inhibit the vaterite formation.^32^

**Figure 5:**
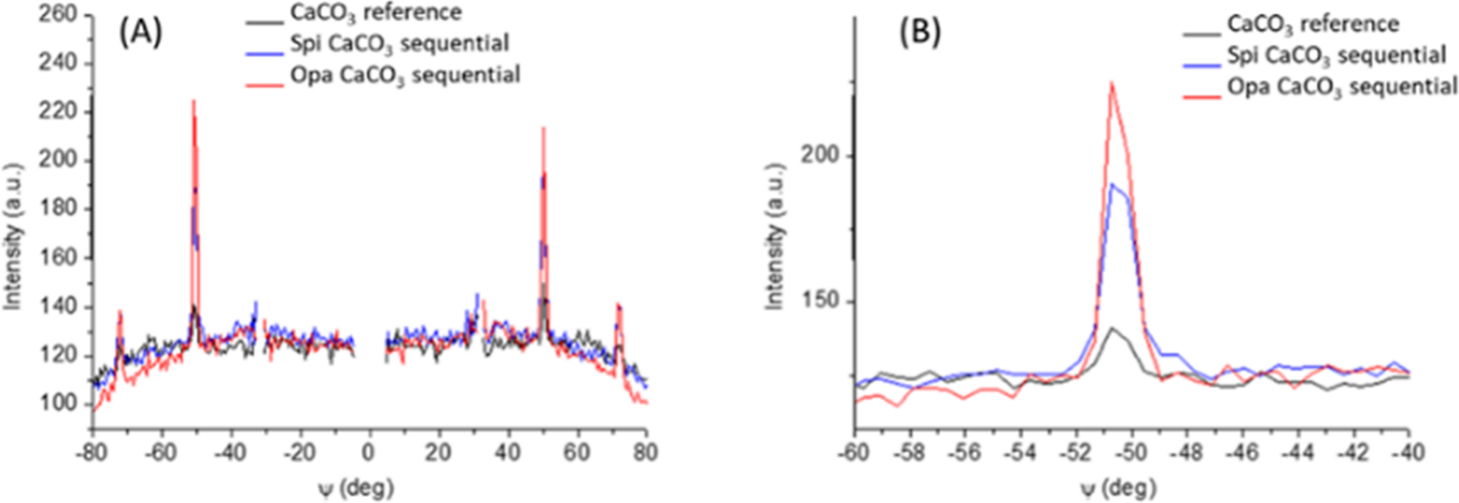
(A) Azimuthal profiles of the (10–2) reflection of calcite extracted from 2D-GIWAXS images of the reference CaCO_3_ sample (black curve from Figure 2E), and sequentially deposited CaCO_3_ on Spi SOM (blue curve from Figure 4C) and on Opa SOM (red curve from Figure 4D) samples. (B) Zoom of one azimuthal peak reported in (A).

The SEM images (Figure 4A,B) indicate that the calcite formation occurs preferentially in the regions where the SOM substrate was present: crystallites are not detected on a smooth surface, associated with the neat silicon wafer, and the EDX mapping does not show the presence of calcium (Figures S6 and S7). Interestingly, the SOMs after the interaction with the CaCO_3_ dispersion (i) lost their layered crystalline structure and (ii) reorganized their distribution of the surfaces. The former is deduced by the lack of low angle diffraction signals in GIWAXS images (Figure S10) and the latter by the OM and SEM images (Figures 4, S6, and S7). This observation suggests that the SOM substrates are not rigid structures, but upon interaction with the CaCO_3_ particles dispersion, they lose the periodic order, partially dissolve, and reaggregate. On the GIWAXS images (Figure 4C,D) the diffraction signals of vaterite reflections are weaker (in the case of Spi SOM) than the corresponding reference samples or completely absent (in the case of Opa SOM), whereas the calcite diffraction spots are not affected by the presence of the SOM. These results indicate that the vaterite phase, dominant in the reference system, is largely suppressed by the presence of the SOM. From these data, it seems that the SOM molecules do not lose the capability to interact with CaCO_3_ particles favoring their crystallization and eventually orientation. Indeed, 2D-GIWAXS data indicate the same arrangement observed for the reference samples, but the SOM presence induces an increase in the ratio of single crystals to polycrystals, as deduced by the increased intensity of the spots compared to the broad contribution, as clearly seen in the azimuthal profiles (Figure 5A,B).

**Figure 6:**
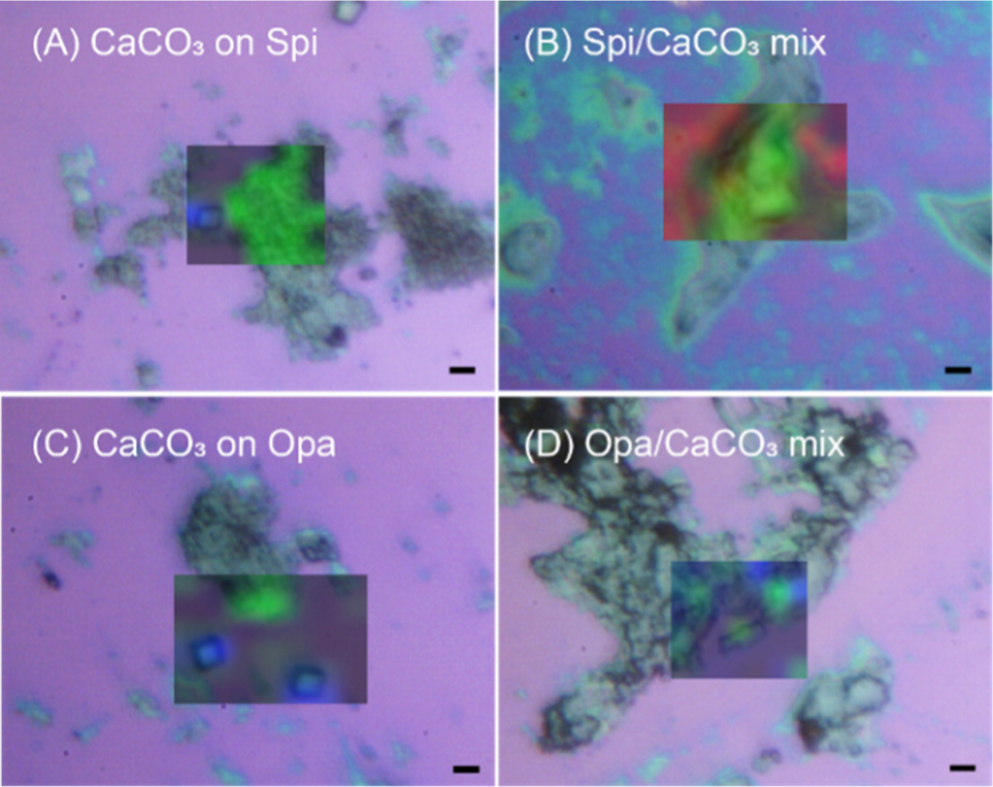
Micro-Raman microscopy analyses on CaCO_3_ deposited on silicon substrates by sequential or mixing experiments showing optical microscopy images and CaCO_3_ phase overlaid color Raman maps. (A,B) CaCO_3_ deposited on the Spi SOM substrate from a CaCO_3_ dispersion (A) and on the silicon substrate from a Spi SOM–CaCO_3_ mixture (B). (C,D) CaCO_3_ deposited on the Opa SOM substrate from a CaCO_3_ dispersion (C) and on the silicon substrate from an Opa SOM–CaCO_3_ mixture (D). In the overlaid color Raman map, green indicates ACC and blue indicates calcite. The red background is due to the silicon substrate. The mineral phases were assigned according with previous studies.^33,34^ Scale bar: 4 μm.

Additional information was obtained from the Raman spectroscopy images (Figure 6). Calcite was colored according to the Eg vibration at about 280 cm^–1^, ACC was colored according to the broad vibration at 1080 cm^–1^, and Si was recognized by the phonon at 520.7 cm^–1^. It is easier than trying to differentiate between the two peaks at around 1086 cm^–1^ narrow for calcite and broad for ACC centered around 1080 cm^–1^. The Raman spectra confirmed the presence of calcite in the samples and also showed the copresence of ACC (Figure 6A,C). The latter was observed in the nonbirefringent aggregates and appeared in association with the SOM matrices. The Raman data did not show relevant differences between the SOM from *S. pistillata* and that from *O. patagonica*. The presence of ACC indicates not only that the crystalline phase formed from ACC,^39^ as expected from the starting condition of supersaturation, but also that the SOM from both species interacts and stabilizes the ACC^40^ since in the control experiments, only crystalline phases were observed (Figure 2). The Raman spectra as well as GIWAX images were collected on dry samples obtained by spin coating. We hypothesize that these dry samples are chemically stable. The observation of ACC indicates that it is in a stable form, able to remain unchanged for a period of months.

In general, the results from this first set of experiments generally agree with what was observed by the Addadi group in a pioneering work using SOM from mollusk shells.^23^ In those studies, a preferential [001] orientation of calcite crystals was observed on a glass substrate where SOM was adsorbed before the crystallization of calcite by the ammonium carbonate vapor diffusion method.^23^ Here, we add the information that the formation of the calcite crystals occurs through an ACC phase that is stabilized by the SOM. Thus, the SOM, when adsorbed on a substrate, stabilizes the ACC phase and influences the orientation of the crystals.

### SOM–CaCO_3_ Mix Chemical System

**Figure 7:**
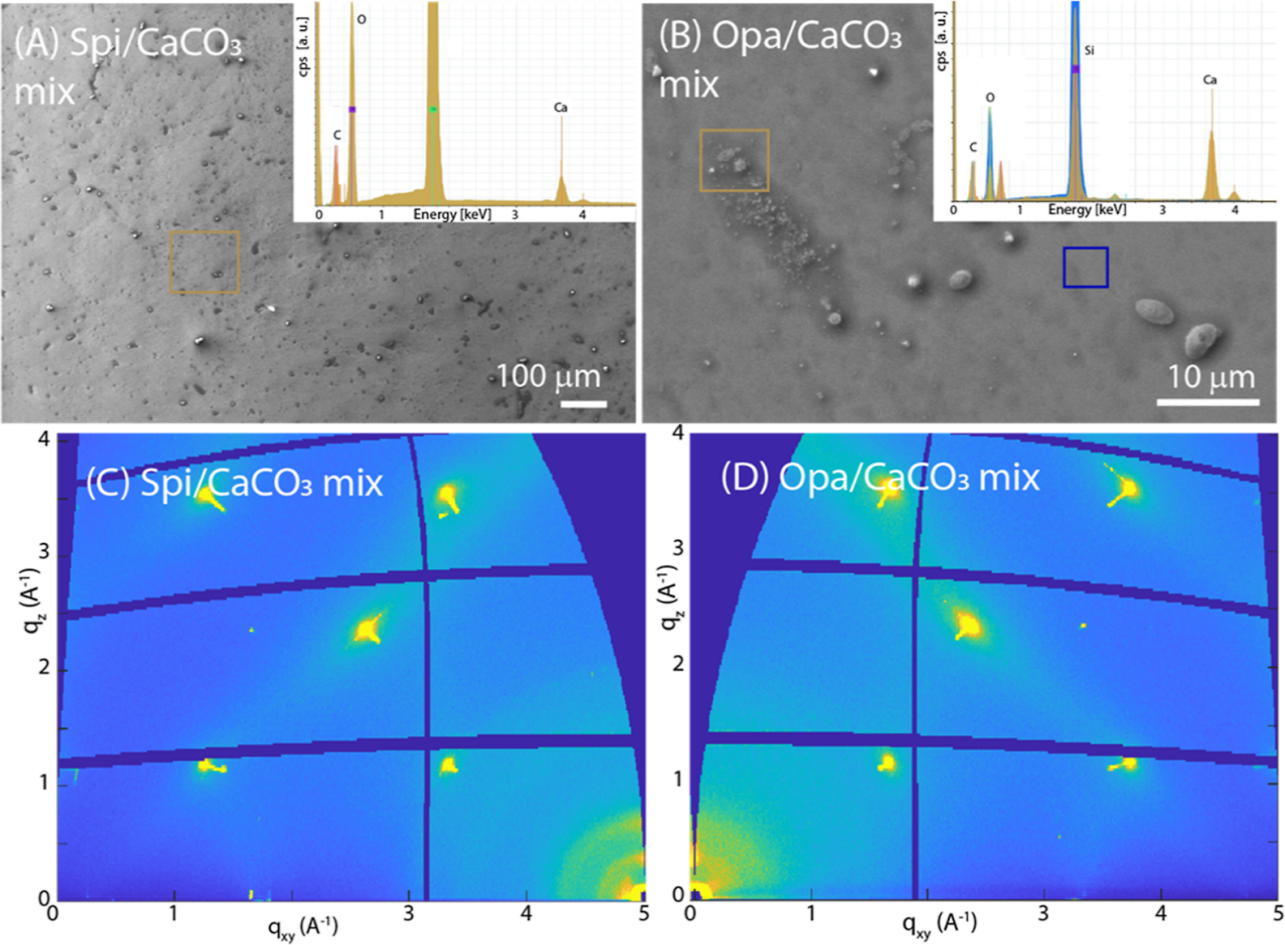
(A,B) SEM images of a CaCO_3_/SOM mix deposited on a silica substrate with SOM from *S. pistillata* or *O. patagonica*, respectively. In the inset, the EDX spectra recorded from the square regions drawn on the SEM images are reported. (C,D) GID images from CaCO_3_/SOM mix deposited on silica substrate with SOM from *S. pistillata* or *O. patagonica*, respectively.

The formation of crystalline faces was not observed when the SOMs were mixed with the CaCO_3_ dispersion before CaCO_3_ deposition on the silicon substrate. Also, the polarization optical microscopy images did not show any birefringence (Figures S4 and S5) and no mineral CaCO_3_ diffraction effect was present in the GIWAXS images (Figure 7), but the EDX mapping showed a strong signal for Ca and C (Figures S6 and S7). In these samples, the Raman microscopy mapping (Figure 6B,D) showed a clear signal from ACC.^57^ It is possible that as the SOM can stabilize ACC,^3^ it represents an aspect of the inhibition of crystallization. In addition to this observation, in the presented case, it seems that the presence of ACC does not affect the SOM arrangement in the case of the Spi SOM, whose peak diffraction positions, i.e., periodicities, widths, and the mosaicity attain to the values of the film obtained without CaCO_3_ present (Figure S11, Table 1). The Opa SOM molecules still self-assemble on the silicon substrate but in a less efficient way, as witnessed by the extremely low scattered signal, which is almost absent for the second peak (Figure S11B). The first peak has a larger periodicity than that determined for the reference sample, indicating a structural change (Table 1).

### General Considerations

The above results, once more, indicate that much of the knowledge on SOM from mollusk shells also applies to coral skeletons, even if the latter does not present a high level of control on the mineral texture. An observation that agrees with a general calcification strategy reported in a recent paper.^58^ However, this study sheds new light on the pioneering research of Weiner and Hood,^59^ and Wheeler et al.^60^ also addressing aspects related to crystallography. We show that SOM macromolecules play diverse roles depending on their assembly/aggregation state. When they are adsorbed onto a template and have an initial structure,^21,22^ they can promote crystal nucleation through the ACC transient phase, but when in solution, they inhibit crystal growth stabilizing the ACC. The initial nucleation model of Weiner et al.,^22^ which was developed from mollusk shell biomineralization, was based on proteins adopting an ordered conformation (the antiparallel β-sheet) and adsorbed on the faces of a substrate (the chitin core). In this “sandwich” model, the carboxylate groups of the side chains of proteins and sulfated polysaccharides contributed to set the conditions for mineral deposition in a heteroepitaxy model.^23^ Later, it was shown that in nacre samples, a gelling state occurs in the organo-mineral assembly,^61,62^ and that the tablet is surrounded by a thin layer of ACC.^63^ The most recent model of CaCO_3_ biomineralization suggests that the mineral deposition occurs by particle attachments of ACC on the mineral surface,^14^ that is present in a gelling environment. On the contrary, in corals’ SOM, no evidence of β-sheet order confirmation was reported, but most SOM identified to have at least one intrinsic disorder region providing a building block for mineral deposition.^32^ Our data suggest that the SOM has a layered structure and that this structural organization is lost when the oriented mineral deposition occurs. However, this layered organization is still present when ACC is dispersed into the SOM. We are not able to assess whether the initial formation of ACC occurs on SOM absorbed on the substrate, but we observe that the crystalline layered assembly of SOM is lost when calcite crystals are formed. This layered state is instead conserved when SOM is mixed in CaCO_3_ particles dispersion. We are confident that this layered structure could also be generated by SOM extracted by other species of corals. Indeed, in our previous research it was observed that the chemical composition of the SOM is quite similar among species, being characterized by a high content of aspartic and glutamic residues and being the proteins glycosylated.^3,32,35,64^

Based on our experimental results, we propose the following model: SOM adsorbed on the substrate initially interacts with the ACC particles and preserves its layered structure; when the crystallization process occurs, the macromolecules of SOM may influence the orientation of the calcite crystals but lose their stratified structure. We can suppose that, being the SOM proteins intrinsically disordered,^32^ the control of crystal orientation, if not governed by the morphology of calcite, could be attributed to geometrical restrains,^65^ differently from what reported for mollusk, where proteins assume a beta structure.^23^ What triggers the transition from the amorphous to the crystalline phase is not determinable in this study. It is also important to consider the formation of calcite instead of aragonite, the mineral phase forming the coral skeleton, as expected since in in vitro experiments aragonite forms at room temperature only in the presence of Mg ions.^66^ The use of Mg ions was avoided to reduce the complexity of the system and considering that the used chemical system is a model for the study of the biomineralization process.^67^

However, on the basis of the presented results, it can be assumed that in the in vivo system, the different macromolecules that make up SOM can be secreted by the cells at different times and spaces, carrying out their activities in the right space and time. This model is in good agreement with the two-step mode of growth proposed by Cuif and co-workers for the biomineralization of corals,^31,68^ which supposes an initial formation of a macromolecular layer on which the oriented deposition of aragonite crystals takes place.

## Conclusions

In this study, we showed that SOM from coral skeleton can assemble in an ordered layered structure and that it can induce the oriented crystallization of calcite. Furthermore, we demonstrated that the layered structure remains even when trapped ACC is present but is lost when ACC crystallizes. This observation is completely new for coral and integrates and enriches the biomineralization model for mollusks proposed by Addadi and Weiner,^2,69^ and Weiner and Traub.^20,22^ We are confident that this approach to the study of coral biomineralization processes will contribute to a significant improvement in the understanding of biomineralization processes, inspiring studies on different substrates and with different SOMs. It will also awaken the interest of researchers in the study of the crystallographic aspects of biomineralization, which still represents one of the most intriguing aspects of biominerals.

## Supporting Information Available

Detailed description of the optimization of the experimental setup, optical microscopy images, sample EDX maps, and additional 2D-GIWAXS data (PDF)
