# Supplementary material for: Assembly of the Intraskeletal Coral Organic Matrix during Calcium Carbonate Formation
Source: Cryst Growth Des. 2023 Jul 15;23(8):5801–11. doi: 10.1021/acs.cgd.3c00401 (PMC10401569; doi:10.1021/acs.cgd.3c00401)
Supplement: Supplementary file 1 — cg3c00401_si_001.pdf [file cg3c00401_si_001.pdf]

Supporting Information

Assembly of the intraskeletal coral organic matrix during calcium carbonate formation

Silvia Milita,[1,*] Tal Zaquin,[2] Simona Fermani,[3,4] Devis Montroni,[3] Iddo Pinkas,[5] Luisa Barba,[6] Giuseppe Falini[3,7,*], and Tali Mass[2,*]

[1]CNR – Institute for Microelectronic and Microsystems, via Gobetti 101, Bologna, 40129, Italy. [2]Department of Marine Biology, The Leon H. Charney School of Marine Sciences, University of Haifa, 3498838, Mt. Carmel, Haifa, Israel. [3]Department of Chemistry 'Giacomo Ciamician', University of Bologna, via Selmi 2, Bologna, 40126, Italy. [4]Interdepartmental Centre for Industrial Research Health Sciences & Technologies, University of Bologna, 40064 Bologna, Italy. [5]Department of Chemical Research Support, Weizmann Institute of Science, 76100, Rehovot, Israel. [6]CNR -Institute of Crystallography, Elettra Synchrotron, I-34100 Trieste, Italy. [7]CNR, Institute for Nanostructured Materials, via Gobetti 101, 40129, Bologna, Italy.

Table 1. Selection experimental set up pag. SI2

Figure SI1. Optical microscope images $CaCO_3$ chemical system SI2

Figure SI2. X-ray diffraction profiles extracted by 2D-GIWAXS images SI3

Figure SI3. Polar map of GIWAXS pattern SI3

Figure SI4. Optical microscope images SOM – $CaCO_3$ *S. pistillata* system SI4

Figure SI5. Optical microscope images SOM – $CaCO_3$ *O. patagonica* system SI5

Figure SI6. SEM images and EDS maps SOM – $CaCO_3$ *S. pistillata* system SI7

Figure SI7. SEM images and EDS maps SOM – $CaCO_3$ *O. patagonica* system SI7

Figure SI8. Series of 2D-GIWAXS images at different incident angle SI8

Figure SI9. 2D-GIWAXS data analysis SI8

Figure SI10. Small angle region of 2D-GIWAXS SOM – $CaCO_3$ *S. pistillata* system SI9

Figure SI11. Small angle region of 2D-GIWAXS SOM – $CaCO_3$ *O. patagonica* system SI9

Table SI1. Observations on the effects of different t_{mix}, t_{cast} and t_{spin} on the precipitation of $CaCO_3$. The optimal experimental condition is texted in italic. The corresponding times are reported in the first column of the table. The presence of crystalline nuclei was evaluated by using an optical microscope having cross-polar.

**Time**	t_{mix}	t_{cast}	t_{spin}
1:00 min	*no crystalline nuclei were observed*	a low amount of $CaCO_3$ deposited	silicon wafer surface appears dry
2:30 min	no crystalline nuclei were observed	*GIWAXS detectable amount of $CaCO_3$ deposited*	*silicon wafer surface is completely dry*
3:30 min	few crystalline nuclei were observed	precipitation of crystals	
5:30 min	precipitation of crystals	massive precipitation of crystals	
10:00 min	massive precipitation of crystals		

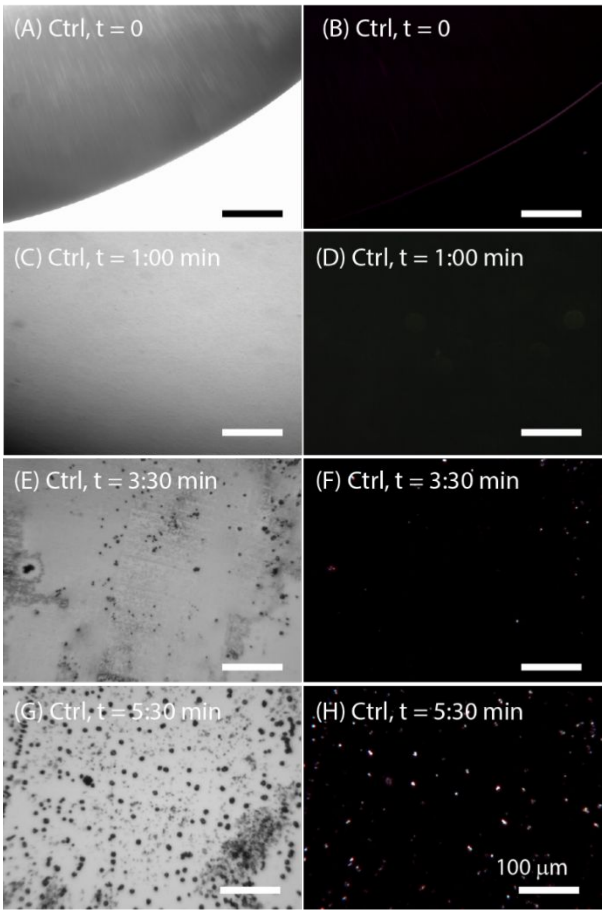

Figure SI1. Optical microscope images (A, C, E and G) and corresponding cross polar images (B, D, F and H) of the time evolution of the $CaCO_3$ formation in a volume of 100 μL deposited on a glass cover slip. Only few times are illustrated respect to the all set of experiments (see Table SI1).

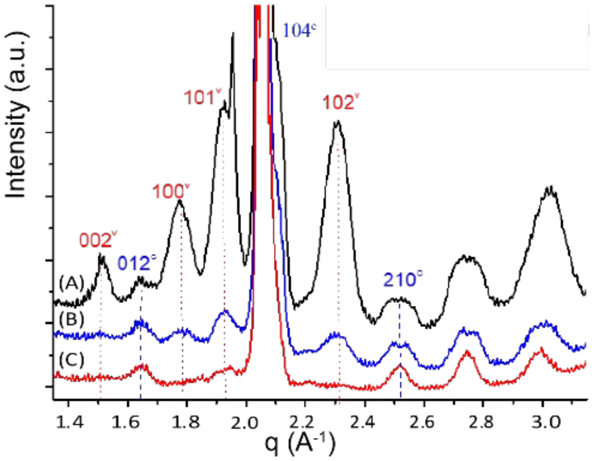

Figure SI2. X-ray diffraction profiles extracted by 2D-GIWAXS images reported in Figure 2C, 5C, and 5D by integrating the intensity in the entire q space. (A) Calcium carbonate formed on the reference substrate of silicon, (B) onto the Spi SOM and (C) onto Opa SOM. The profiles are vertically shifted for the sake of clarity. The diffraction peak Miller indices are indicated for calcite (apex c) and vaterite (apex v).

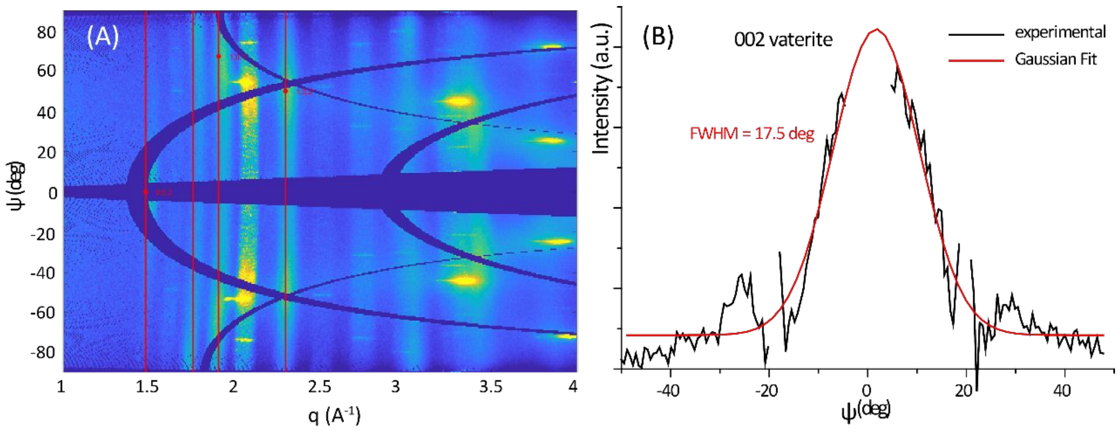

Figure SI3. (A) Polar map obtained from the 2D-GIWAXS of $CaCO_3$ film (reference sample) reported in Figure 2C. The red lines indicate the distribution of intensity along the azimuth angle (ψ) of the diffraction peaks of vaterite. (B) Azimuthal profile of the (200 vaterite, black line) reflection with gaussian fit (red line).

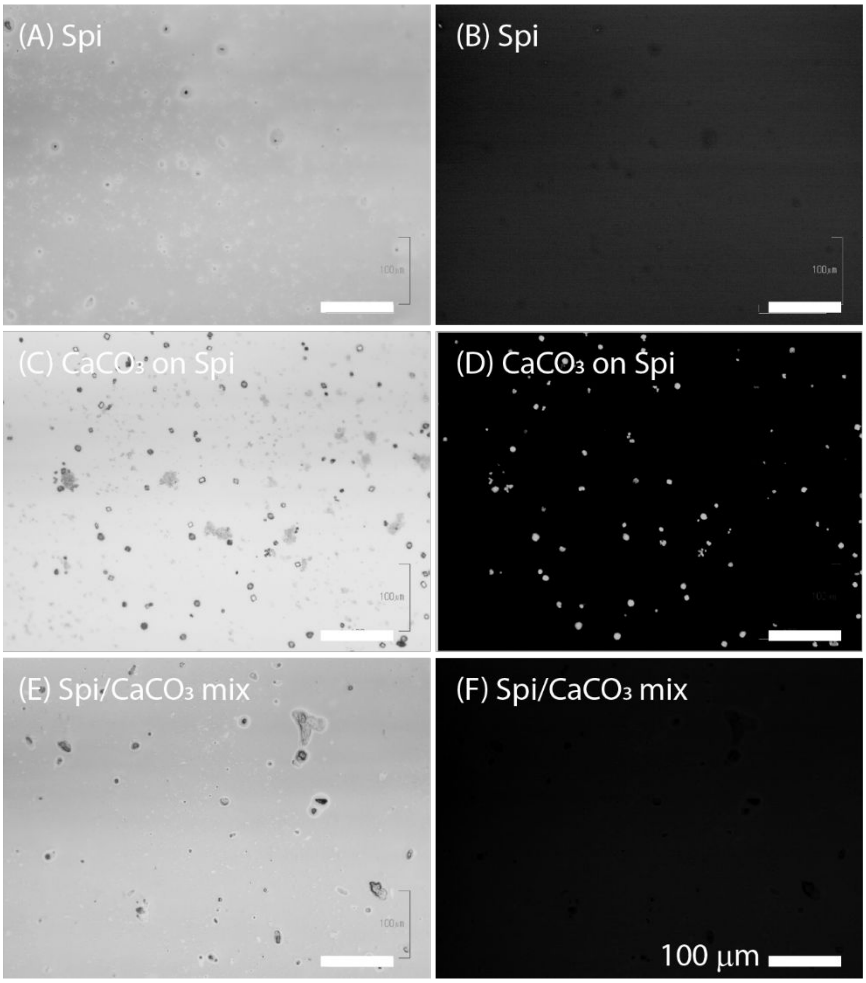

Figure SI4. (A) and (B) report OM image and corresponding birefringent image under cross-polar, respectively, of the SOM from *S. pistillata* deposited on the silicon wafer. (C) and (D) report OM image and corresponding birefringent image under cross-polar, respectively, of the $CaCO_3$ material deposited on the SOM substrate. (E) and (F) report OM image and corresponding birefringent image under cross-polar, respectively, of the $CaCO_3$/SOM material deposited from the SOM/$CaCO_3$ dispersion mixture.

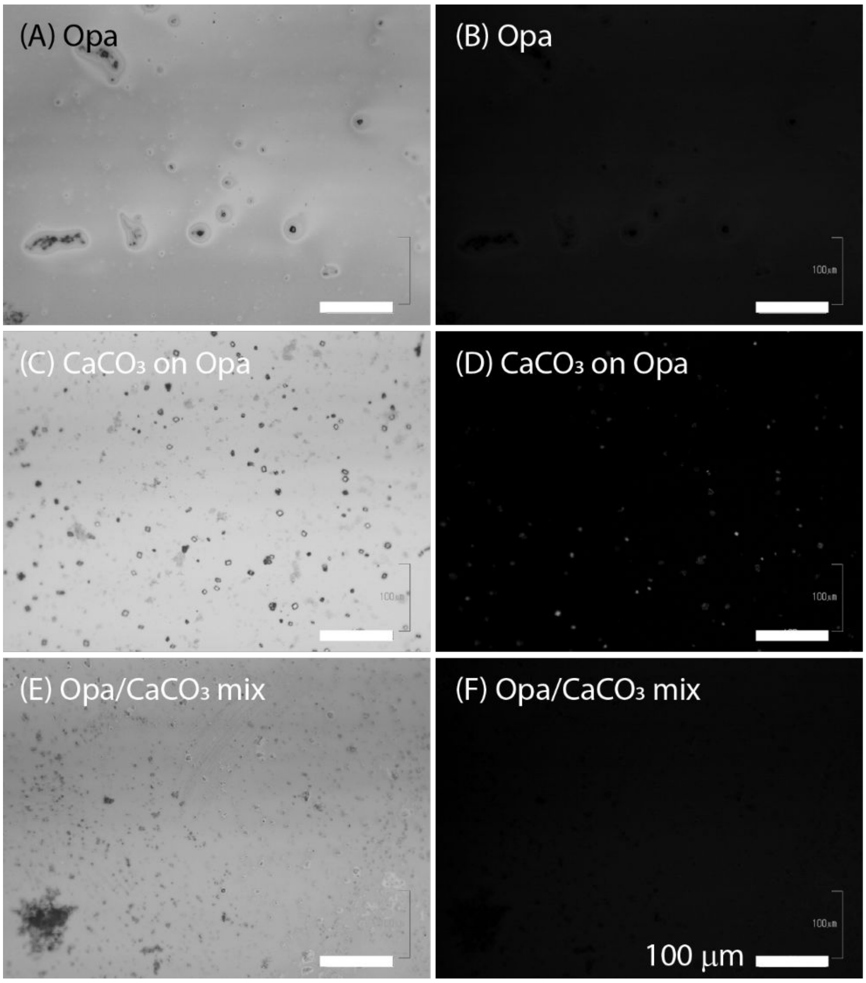

Figure SI5. (A) and (B) report OM image and corresponding birefringent image under cross-polar, respectively, of the SOM from *O. patagonica* deposited on the silicon wafer. (C) and (D) report OM image and corresponding birefringent image under cross-polar, respectively, of the $CaCO_3$ material deposited on the SOM substrate. (E) and (F) report OM image and corresponding birefringent image under cross-polar, respectively, of the $CaCO_3$/SOM material deposited from the SOM/$CaCO_3$ dispersion mixture.

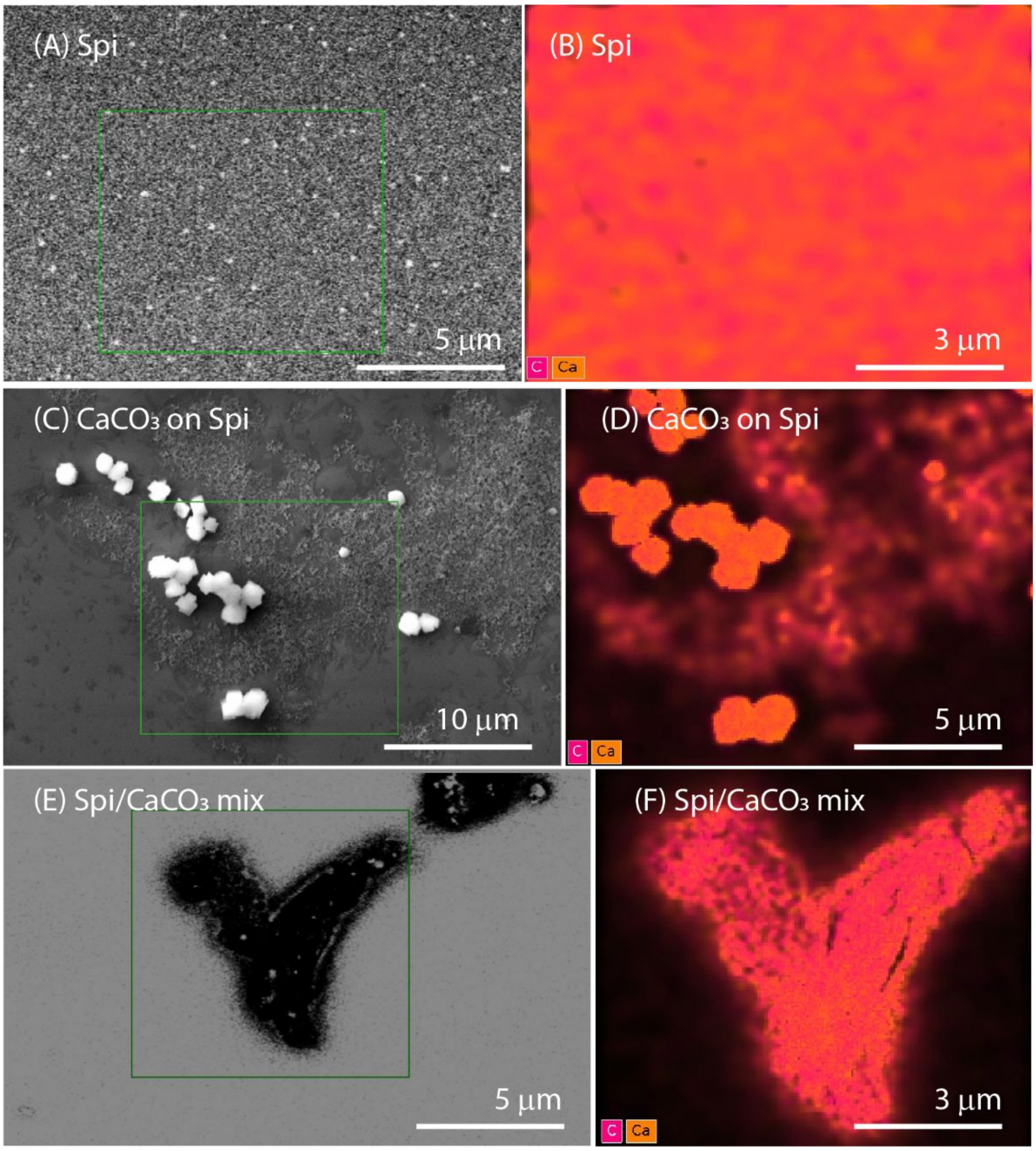

Figure SI6. (A) and (B) report SEM and EDS map of the surface inside the green square, respectively, of the SOM from *S. pistillata* deposited on the silicon wafer. (C) and (D) report SEM and EDX map of the surface inside the green square, respectively, of the $CaCO_3$ material deposited on the SOM substrate. (E) and (F) report SEM and EDX map of the surface inside the green square, respectively, of the material deposited from the SOM/$CaCO_3$ dispersion mixture.

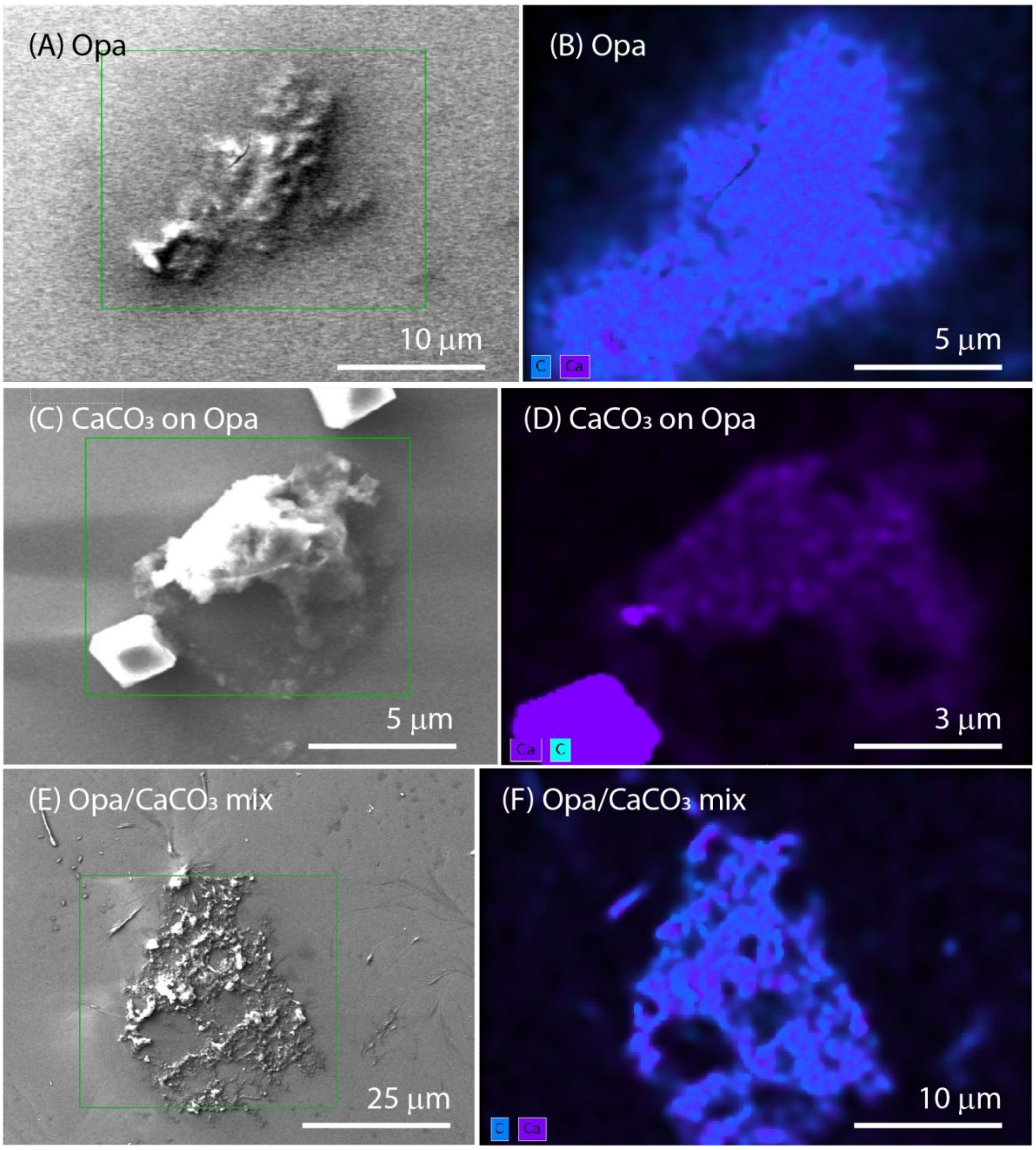

Figure SI7. (A) and (B) report SEM and EDS map of the surface inside the green square), respectively, of the SOM from *O. patagonica* deposited on the silicon wafer. (C) and (D) report SEM and EDS map of the surface inside the green square, respectively, of the $CaCO_3$ material deposited on the SOM substrate. (E) and (F) report SEM and EDS map of the surface inside the green square, respectively, of the material deposited from the SOM/$CaCO_3$ dispersion mixture.

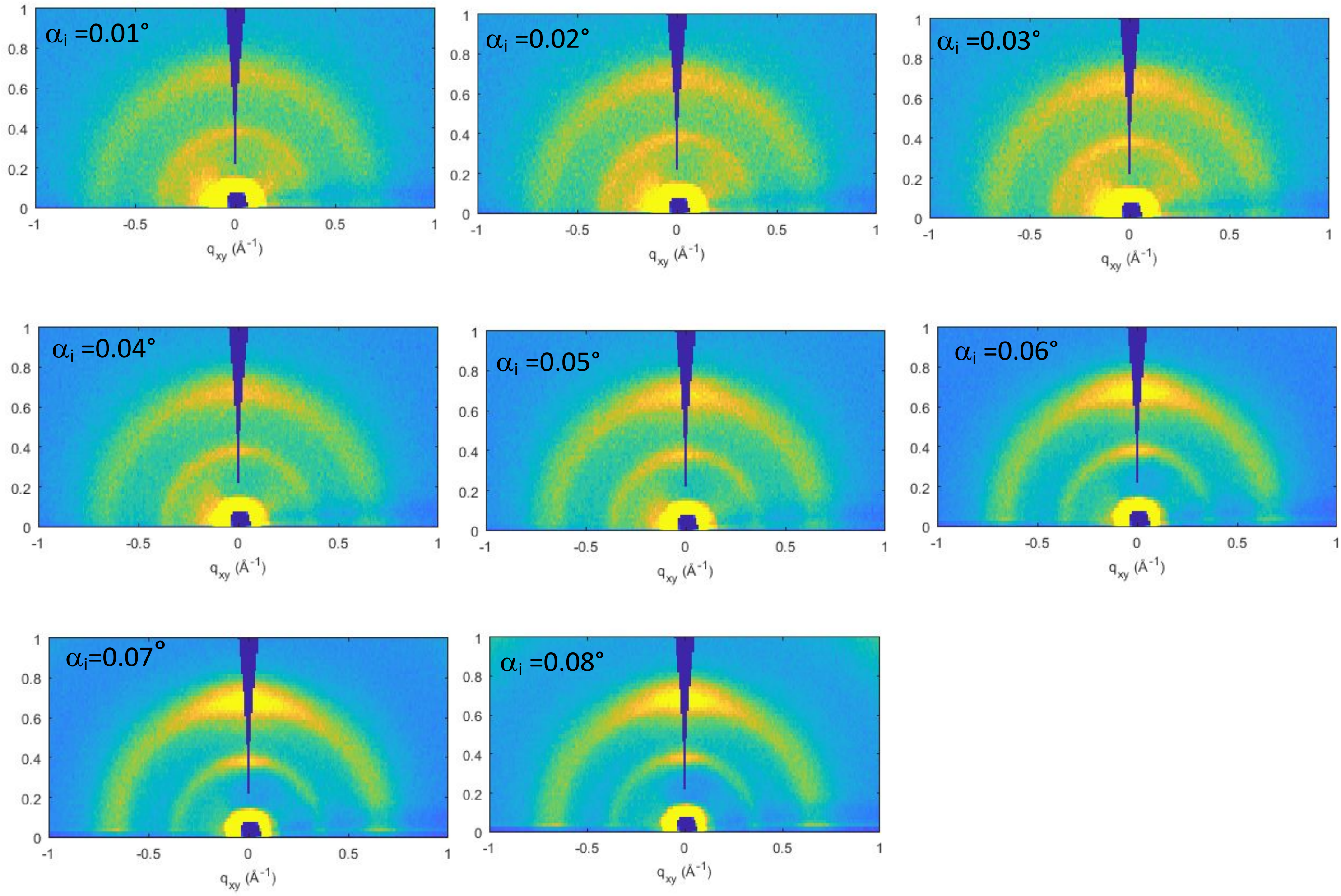

Figure SI8. Series of 2D-GIWAXS images of Spi deposited on silicon substrate recorded at different incident angle (αi).

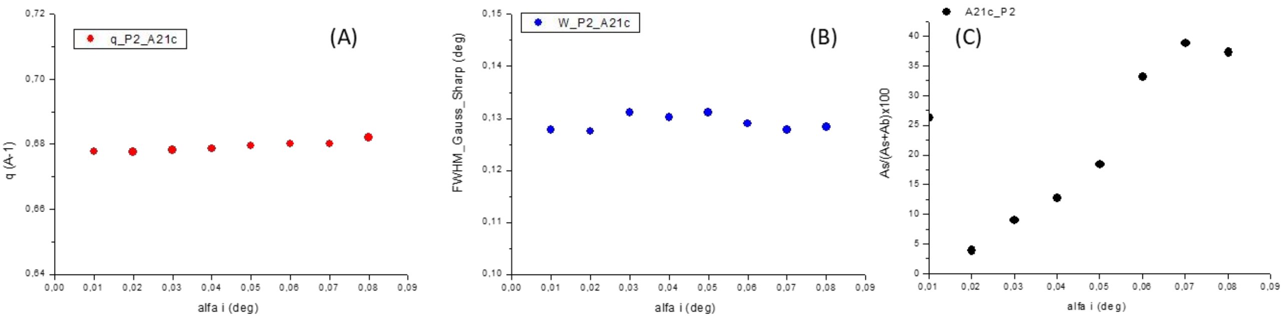

Figure SI9. (A) q, (B) FWHM and (C) As/(As+Ab) profiles of the second reflection extracted by 2D-GIWAXS of Fig. SI7.

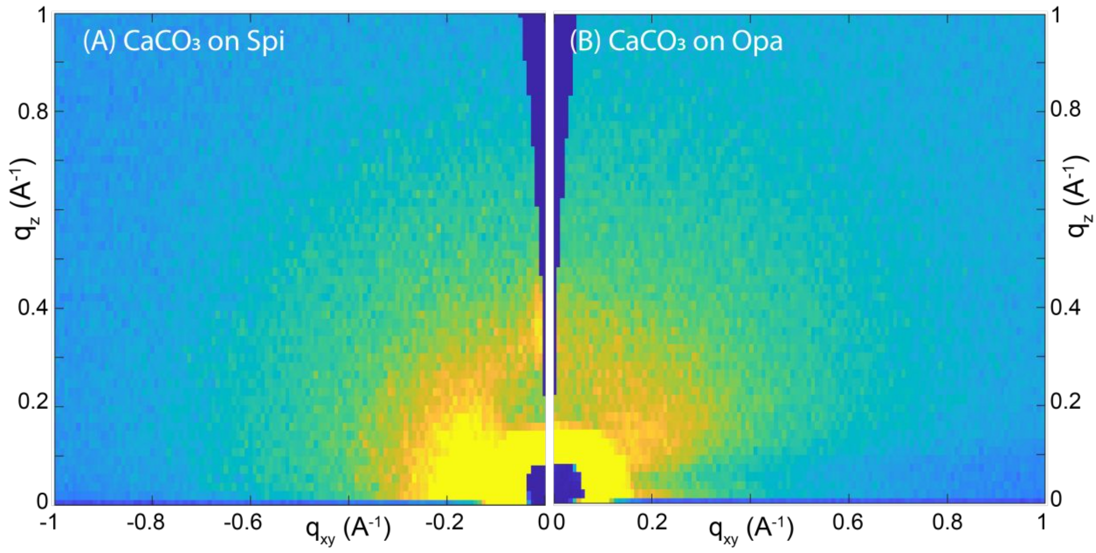

Figure SI10. Small angle region of 2D-GIWAXS of Spi SOM + $CaCO_3$ (A) and Opa SOM + $CaCO_3$ (B) samples from the sequential experiments.

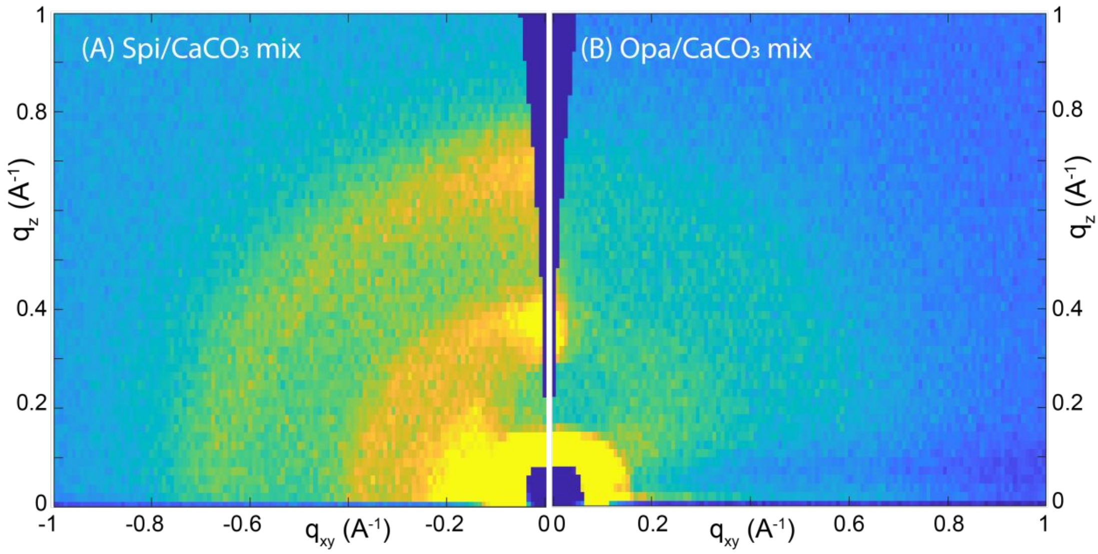

Figure S11. Small angle region of 2D-GIWAXS of Spi SOM+ $CaCO_3$ (A) and Opa SOM + $CaCO_3$ (B) samples from the mix experiments.
